# Erythropoietin; a review on current knowledge and new concepts

**DOI:** 10.12861/jrip.2013.38

**Published:** 2013-10-10

**Authors:** Mohamad-Reza Tamadon, Seyed Seifollah Beladi-Mousavi

**Affiliations:** ^1^Department of Internal Medicine, Semnan University of Medical Sciences, Semnan, Iran; ^2^Department of Internal Medicine, Ahvaz Jundishapour University of Medical Sciences, Ahvaz, Iran

**Keywords:** End-stage renal disease, Chronic kidney disease, Erythropoietin, Kidney transplantation

## Abstract

Chronic kidney disease (CKD) is a worldwide health problem. However, despite to new routes of dialysis, mortality and morbidity is high. One of the most common symptom of CKD is anemia, especially is more obvious in stages 3 and 4. In this review, we compared the effects of erythropoietin and anemia correction on kidney function (GFR) by investigating in various studies. Despite extensive studies in this category, still we do not sure about the effects of erythropoietin and anemia correction on the glomerular filtration rate.

**Implication for health policy/practice/research/medical education:**

It seems that the treatment of anemia by recombinant human erythropoietin in chronic kidney disease, when it accompanied by control of metabolic complications, can improve anemia, however more human studies needs to find the effect of erythropoietin on kidney function.

## Introduction

Chronic kidney disease (CKD) is a serious problem in the general population. There is a growing trend in prevalence and incidence of renal failure in the United States (1). In this review, we compared the effects of erythropoietin and anemia correction on kidney function investigated in various studies. Annual increase in incidence of end-stage renal disease (ESRD) resulted in increasing the number of patients who received kidney transplantation, which was increased survival rate of patients. Despite efforts for treating the ESRD and improve­ment of dialysis quality, still the disease have high mortality and morbidity, and 1, 2 and 5 years survival rate after dialysis have been reported in these patients 81%, 65%, 34% respectively (2). By initiation of different stages of CKD which resulting from decreasing of kidney function, some complications such as uremia, increased of volume, electrolyte disorders and anemia appear and maybe health threatening and can even lead to death if are not properly treated. In other words, the main cause of replacement therapy (dialysis or kidney transplantation) is inability in treating of renal failure complications by drug therapy (3). One of the most important complications of CKD is anemia. Production of red blood cells in the bone marrow controlled by erythropoietin, a glycoprotein hormone which secreted from capillary epithelial cells around the renal tubules; also a few amounts of erythropoietin are produced in liver cells (4).

In the absence of erythropoietin, precursor cells of red blood cells in bone marrow are destroyed (apoptosis). By loss of tissues, around the kidney tubules and its failure, amount of erythropoietin production reduced and anemia occurs (4). In several studies which was conducted so far, it was not concluded that treatment of anemia caused by CKD with recombinant human erythropoietin could improved the renal function and reduced need to dialysis (3-5). Some studies on rats have shown that treatment of anemia due to CKD resulted in more glomeruli damages and exacerbation of hypertension (6-8).

We previously showed that, mean of creatinine level and mean of 1/Cr value creatinine level in ten time monthly measurements (three months before and six months after intervention) and also in four three-month period included three-month period before intervention, time of intervention, the first and second quarter after intervention were not statistically significant (9). Indeed most of investigations referred to this subject that erythropoietin has not any effect on renal function. Although in some cases it has not been firmly confirmed and it needs further studies. Chandra *et al*. was evaluated the relationship of serum erythropoietin (EPO) levels to hematocrit and glomerular filtration rate (GFR) in patients with CKD. The EPO levels was measured by radioimmunoassay in 119 blood samples from 48 patients obtained over a period of up to 5 years. Hematocrit values correlated significantly with the GFR, but serum EPO levels did not change with a decline in the GFR. Significant anemia was noted only when the GFR fell below 20 ml/min/1.73 m^2^. The results of this study suggest that the tissue oxygenation-EPO-hematocrit feedback mechanism operates at a lower set point in patients with CKD in comparison with normal subjects (10). Anemia is a major cause of the decline in exercise capacity seen in these patients. Clyne *et al*. examined the effects of EPO treatment in 12 pre-dialytic uremic patients (EPO group with mean age of 46±12 years including 6 men). They found a significant correlation between the increase in total hemoglobin and the increase in exercise capacity in the EPO-treated group (11). Recent publications have suggested reno-protective actions for EPO in certain models of acute kidney injury in rats. In a recent study by Rafieian-Kopaei *et al*., the effects of erythropoietin on amelioration of gentamicin-induced renal toxicity was investigated by biochemical and histopathological indices (12). The results of this study suggest EPO as a promising reno-protective drug to prevent or attenuate gentamicin-induced tubular damage and introduce a novel therapeutic strategy for patients with this kind of kidney disease. However, there are still needs for studies on the mechanisms which are involved in these protective actions (12,13). The weight of clinical evidence indicates that erythropoietin exerts neither a beneficial nor a deleterious effect on the progression of renal impairment in patients with CKD (Level II Evidence, 6 small randomized controlled trials; clinically relevant outcomes; inconsistent effects). Cumulative renal survival, derived from the time it took baseline plasma creatinine concentration to double, was significantly better in the treated group than in the untreated anemic group, but it was not different from that in untreated non-anemic controls. Dialysis was commenced in 33%, 65% and 37% of patients, respectively. The improvement in cumulative renal survival in the EPO-treated group was attributable solely to improved renal survival in non-diabetics. It was concluded that reversal of anemia by EPO retards the progression of renal failure, especially in non-diabetic patients (they speculated that this was due to prevention of renal tissue hypoxia). Using plasma creatinine, reciprocal plasma creatinine or creatinine clearance to assess progression of renal insufficiency, other prospective studies including an additional three randomized, double-blind, placebo-controlled trials have not observed any significant effect of erythropoietin on renal function. The use of plasma creatinine or creatinine clearance as an index of renal function is a major limitation of all of these studies, particularly since erythropoietin may have significant effects on appetite and muscle metabolism. A recent retrospective cohort study has also suggested that erythropoietin treatment may slow the progression of renal failure. In this study, the authors compared 20 patients with CKD who were treated with erythropoietin with 43 patients who had a similar degree of renal failure but who were less anemic and thus did not receive erythropoietin. The rate of decline of creatinine clearance did not change over time in the control group, whereas in the treated group, it was significantly slower after EPO treatment had been started. Of the 6 RCTs published to date, 5 trials have found no significant effect of EPO administration on the progression of CKD (14). Ble *et al*. tested the hypothesis that the age-related decline in kidney function is associated with an increased prevalence of anemia and that such an increase is accompanied by a concomitant decrement in erythropoietin levels (15). However, after controlling for age, diseases, and other important confounders, and compared with participants with creatinine clearance (CrCl) higher than 90 ml/min, only participants with CrCl of 30 mL/min or lower had a significantly higher prevalence of anemia. Moreover, in the adjusted analysis, no significant trend toward an increase in the prevalence of anemia with reduction of renal function among subjects with mild to moderate CrCl was found (15,16).

A European clinical trial demonstrated that erythropoietin therapy resulted in a greater reduction in exposure to allogeneic blood during orthopedic surgery in patients who donated autologous blood and were anemic (hematocrit, <39%) than in placebo-treated patients (17). However, this trial included supplemental iron administered intravenously as well as orally.

## Conclusion

It seems that the treatment of anemia by recombinant human erythropoietin in chronic kidney disease, when it accompanied by control of metabolic complications, can improve anemia but has not any effect on reduction of renal function rate. For confirming of erythropoietin effect on renal function, we need a study with larger samples size and longer period of time.

## Acknowledgments

We thank Mr Mehrdad Zahmatkesh for helping in writing this article.

## Authors’ contributions

Main draft write up and editing by MRT. Important intellectual content and critical revision by SSBM.

## Conflict of interests

The author declared no competing interests.

## Ethical considerations

Ethical issues (including plagiarism, informed consent, misconduct, double publication and redundancy) have been completely observed by the authors.

## Funding/Support

None declared.
